# Probing the Limits of Mechanical Stability of the Mesoporous Metal–Organic Framework DUT-76(Cu) by Hydrocarbon Physisorption

**DOI:** 10.1021/acsami.5c00164

**Published:** 2025-04-08

**Authors:** Kai Konowski, Volodymyr Bon, Martin A. Karlsen, Martin Etter, Nadine Bönisch, Ankita De, Stefan Kaskel

**Affiliations:** † Chair of Inorganic Chemistry I, Technische Universität Dresden, Bergstraße 66, Dresden 01069, Germany; ‡ P02.1 Beamline, PETRA III Synchrotron, DESY, Notkestraße 85, Hamburg 22607, Germany

**Keywords:** adsorption, DUT-76, total scattering, *n*-alkanes, *n*-alkenes, crystal size, metal−organic frameworks

## Abstract

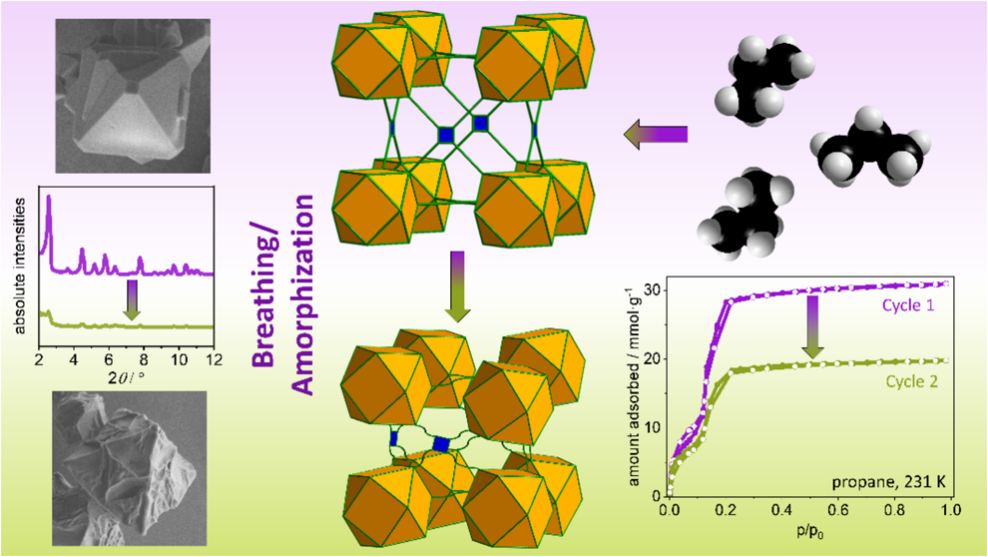

The mechanical robustness of MOFs is crucial in most adsorption-related applications. Herein, we investigated the interaction of the mesoporous metal–organic framework DUT-76(Cu) with various C1–C4 hydrocarbons at their boiling points. During adsorption, the pore structure partially collapsed into an amorphous phase while retaining a residual porosity. We employed a combination of multicycle physisorption experiments using different hydrocarbons (methane, ethane, ethylene, propane, propylene, *n*-butane, and 1,3-butadiene) along with X-ray diffraction, scanning electron microscopy, and total scattering to examine this transition. This methodology allowed us to gain a comprehensive understanding of the effects on the crystal structure, local structure, and macroscopic behavior of the material. Furthermore, we identified specific correlations among the chain length, number of double bonds, and adsorption/desorption cycle stability, which are influenced by adsorption-induced stress. These multicycle adsorption experiments served as semiquantitative tools for assessing the mechanical stability of mesoporous frameworks.

## Introduction

Within the field of porous materials, metal–organic frameworks (MOFs) rank among the most porous yet versatile compounds.^1−3^ This is due to their unique modular construction based on organic linkers and inorganic metal nodes and the effect of the often particularly large inner surface, making them highly advanced adsorbents. Gas adsorption in MOFs can be accompanied by unique and often counterintuitive structural changes within the porous framework due to the complex interplay of inter- and intramolecular interactions between the adsorbate and the adsorbent.^4,5^ This flexibility also sets them apart from other classes of porous materials, such as porous carbons or zeolites, and leads to a variety of analytic methods that provide deep insights into the underlying mechanisms on a molecular scale.^5−9^

Besides these molecular-level constructions and interactions, crystal size has been shown to have an impact on the adsorption behavior of MOFs as well.^10−14^ In many cases, the decrease in crystal size, and thereby the decreased ratio of the inner surface compared to the outer surface of the crystals, leads to the stabilization of a metastable open pore phase due to the lack of nucleation sites required for the phase transition.^15^ This influences the thermodynamics and kinetics of the physisorption process and the possible underlying flexible transition.^11,15^ Various groups have found that a smaller crystal size in ZIF-8 increases the gate-opening penalty and therefore overall decreases the flexibility of this system.^13,16,17^ On the other hand, larger crystals often show more internal strain due to the larger number of differently oriented domains within the crystal.^11,18^ This is also reflected by computational studies, which suggest that the breathing transition of MIL-53(Al) can occur via a layer-by-layer mechanism as well as one involving multiple discrete nucleation points, depending also on external pressure.^14^ This leads to altered kinetics of the phase transition, as in MIL-53(Al), a slower breathing transition with increasing crystal size, but also a lack of phase transition in submicron-sized crystals.^19^ The altered kinetics resulting from changes in the crystal size have been utilized to boost the dynamic separation of ethylene and ethane by increasing the crystal size of the ZnAtzPO_4_ framework.^20^ In general, the crystal size and morphology need to be acknowledged as factors that can influence the physical properties of MOF materials, but can also be a tool to design new MOF materials for specific applications.^10,12,21^

While adsorbing and desorbing gas molecules, MOFs are exposed to an adsorption/desorption stress, which may cause reversible or irreversible framework deformation and may lead to changing its crystallite surface texture, causing breaks or defects, replacing solvent molecules from the synthesis or accumulating in the pore after repeated exposure.^22^ This is often accompanied by a decrease in the crystal size due to the fracture of larger crystals until a certain finite value is reached, an increase in gate-opening pressure and a less steep opening slope, a decrease in gate-closing pressure, and a smaller total pore volume (TPV).^22^ These structural changes can be at least partially understood as a material response to adsorption-induced stress exerted from a fluid adsorbate onto its solid surface.^23,24^ Such a phenomenon can be termed “adsorption milling”. Adsorption stress is also observed in other porous materials such as porous carbons.^25,26^ Gor and Neimark modeled the adsorption-induced deformations in mesoporous silica using the Derjaguin–Broekhoff–de Boer theory for capillary condensation and quenched solid density functional theory, indicating the advantages and disadvantages of each material with particular pore size.^27^ Coudert and Neimark developed a stress-based model for breathing crystalline porous solids.^28^ In terms of experimental methodology, mechanical stress is often evaluated using mercury intrusion,^29,30^*in situ* high-pressure powder X-ray diffraction (PXRD) experiments,^31^ or *in silico* prediction.^32,33^ However, these techniques have certain limitations. For example, in the case of rigid mesoporous frameworks, which are often quite sensitive to ambient conditions, irreversible transitions are observed at relatively low pressures. In such cases, more precise and sensitive techniques must be developed.

**Figure 1:**
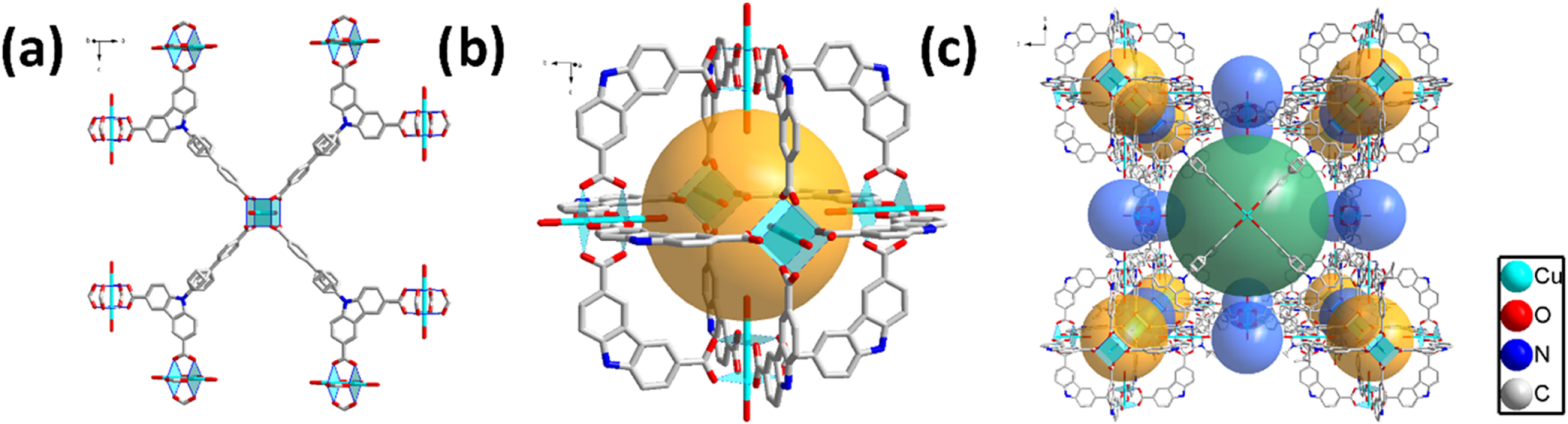
Crystal structure of DUT-76(Cu): (a) copper paddle wheel and organic ligand H_3_CBCDC; (b) carbazole-based metal–organic polyhedron (MOP); and (c) crystal structure of DUT-76(Cu) showing three different pores.

In 2015, Stoeck et al. published a new mesoporous rigid MOF, DUT-76(Cu), with an exceptionally high apparent surface area of 6344 m^2^·g^–1^.^34^ A characteristic feature of this material is the utilization of a carbazole-based cuboctahedral 12-connecting metal–organic polyhedron (MOP), which is also present in other DUT materials acting as a well-defined building block for the construction of MOFs with high porosity and a high concentration of open metal sites.^35−37^ Additional open metal sites were introduced using tritopic linker H_3_CBCDC (9-(4′-carboxy-[1,1′-biphenyl]-4-yl)-9H-carbazole-3,6-dicarboxylic acid) in the construction of the framework, in which carboxylate groups at the biphenyl moiety form an additional copper(II) paddle wheel connecting four MOPs, yielding a (4,12)-connected network with a four, 12-connected (ftw) topology (see Figure 1).^34^ Previously, we found that DUT-76(Cu) had an exceptional capacity for high-pressure ethylene storage at 298 K.^34^

In the following, we use DUT-76(Cu) as a model system and propose an experimental methodology for assessing the mechanical stability of mesoporous MOFs by applying adsorption/desorption stress upon the physisorption of hydrocarbons. The physisorption of selected alkanes and alkenes at their standard boiling points in combination with *in situ* and *ex situ* PXRD analysis, total scattering, and scanning electron microscopy (SEM) imaging are used to fine-tune the adsorption/desorption stress, leading to guest-dependent irreversible contraction of the structure.

## Experimental Section

### Synthesis of Microcrystalline Powder of DUT-76(Cu)

The synthesis of the organic linker and the solvothermal synthesis of DUT-76(Cu) as a microcrystalline powder was performed according to a literature procedure.^34^ In a 100 mL screw cap bottle, 361.1 mg of 9-(4′-carboxy-[1,1′-biphenyl]-4-yl)-9H-carbazole-3,6-dicarboxylic acid (0.80 mmol, 1 equiv) and 309.2 mg of copper(II) nitrate trihydrate (1.28 mmol, 1.6 equiv) were dissolved in 72 mL of mixture dry DMF and dry EtOH (1:1), and 3.2 mL of acetic acid (3.36 g, 56.00 mmol, 70 equiv) was added. The compounds were dissolved by ultrasonication for 5 min, and the solution was transferred into 8 Pyrex tubes and heated to 80 °C for 2 days. The blue crystals were washed with a fresh solvent mixture (dry DMF and dry EtOH, 1:1) 5 times and then exchanged with dry acetone. The desolvation of DUT-76(Cu) was achieved by using a supercritical point dyer. This procedure yielded 262 mg (0.5 mmol, 61%) of desolvated DUT-76(Cu). The sample purity, adsorption properties, and crystal size distribution of the samples were determined using PXRD, nitrogen physisorption, and SEM measurements.

### Desolvation of Microcrystalline Powder of DUT-76(Cu)

The procedure for the desolvation of DUT-76(Cu) was shown to be critical for the stability and physisorption behavior of the material. Specifically, fast gas release for flushing, release of excess CO_2_ from the autoclave, and fast heating to generate supercritical CO_2_ have been shown to lead to the degradation of the material. Therefore, we aimed to provide detailed information on desolvation and thermal activation. Supercritical drying of the MOF powder after synthesis was performed using a Jumbo Critical Point Dryer (13200J AB, SPI Supplies). The MOF was suspended in dry acetone and transferred to the glass filters of the dryer. The autoclave was filled with liquid CO_2_ at 290 K and 7.5 MPa. By opening the valve at the bottom of the autoclave, the remaining acetone was removed and fresh liquid CO_2_ was refilled from the top of the autoclave. This procedure was repeated 8 times per day for 2 days until no acetone traces were observed in the dry ice at the outlet. In the following step, the valve from the gas cylinder to the dryer was closed, and half of the liquid CO_2_ in the autoclave was released. Then, all valves of the autoclave were closed, and the temperature was increased to 313 K to convert CO_2_ into the supercritical state. The pressure in the autoclave was maintained at 9 MPa during heating. After equilibration of the system at 313 K overnight, supercritical CO_2_ was released through the bottom valve for 12 h. The autoclave was opened, as the pressure was reduced to ambient pressure and the dried material was directly brought into an argon-filled glovebox. Each further handling was carried out exclusively under an inert atmosphere. Subsequently, for supercritical drying, the material was heated up to 453 K in a dynamic vacuum for 24 h to remove the remaining guest molecules from the pores.

### Physical Methods

*Ex situ* PXRD patterns were collected in transmission geometry on a STOE STADI P diffractometer equipped with a line-focus Cu X-ray tube operated at 40 kV/30 mA, a focusing Ge (111) monochromator (λ = 0.15405 nm), and a MYTHEN (DECTRIS) detector. A scan speed of 120 s/step and a detector step size of 2θ = 6° were used in the measurements.

Low-pressure (*p* < 110 kPa) volumetric adsorption experiments were carried out using a BELSORP-max instrument (Microtrac MRB), and the measuring routine of BELSORP-max control software was used. The dead volume was routinely determined using helium at 298 K before each measurement as well as between each adsorption/desorption cycle of the cycling experiments. A closed-cycle helium cryostat was used for cooling. Cryostat DE-202AG was operated using a temperature controller LS-336 (LAKE SHORE), and the heat produced by the cryostat was removed from the system by a water-cooled helium compressor ARS-2HW. The sample was placed in a custom-made cell consisting of a 3 cm-long rod-shaped copper cell of 1 cm diameter, sealed from the exterior with a copper dome, insulated by dynamic vacuum (*p* < 10^–4^ kPa), and connected to a BELSORP-max adsorption instrument with a 1.5 mm copper capillary.

*In situ* PXRD experiments on DUT-76(Cu) in parallel with the adsorption and desorption of hydrocarbons at their boiling points were conducted according to a previously published setup and procedure.^9,38^ A customized setup based on a laboratory powder X-ray diffractometer Empyrean-2 (PANALYTICAL GmbH) equipped with a closed-cycle helium cryostat (ARS DE-102) and a home-built X-ray transparent adsorption cell connected to a volumetric adsorption instrument BELSORP-max (Microtrac MRB) was used. The TTL trigger was used to establish the communication between BELSORP-max and Empyrean, and to ensure the measurement of the adsorption isotherm and PXRD pattern data collection in a fully automated mode at the predefined points of the isotherm. The parallel linear Cu Kα_1_ beam, obtained by using a hybrid 2xGe(220) monochromator, 4 mm mask, and primary divergence and secondary antiscatter slits with a 1/4° opening, were used for data collection. A Pixcel-3D detector in 1D scanning mode (255 active channels) was used. Diffraction experiments were performed using ω–2θ scans in transmission geometry in the range of 2θ = 3–70°.

SEM images of DUT-76(Cu) were taken with secondary electrons using a HITACHI SU8020 microscope at an acceleration voltage of 1.0 kV and a working distance of 15.2–16.4 mm. The sample was prepared under an argon atmosphere on an inert sample holder. As the sample showed degradation when prepared directly on a sticky carbon pad, powdered samples were prepared on a dry silicon wafer attached to a sample holder with a sticky carbon pad. The residual powder was blown away with argon and a magnet to prevent contamination on the device. The samples were then transferred to the device under argon atmosphere on an inert sample holder. For crystal size determination, at least 10 images from each sample were analyzed using the ImageJ Software package.

Thermogravimetric analysis (TGA) was carried out in argon using a NETZSCH STA 409 thermal analyzer at a heating rate of 5 K·min^–1^. An air-sensitive MOF sample was prepared in an Ar-filled glovebox and inserted into the instrument with little exposure to ambient conditions.

The total X-ray scattering data were collected at the Powder Diffraction and Total Scattering Beamline, P02.1, PETRA III, Deutsches Elektronen-Synchrotron (DESY).^39^ An X-ray wavelength of λ = 0.0207361 nm was used, and the data were collected using Varex XRD 4343CT (150 × 150 μm^2^ pixel size, 2880 × 2880 pixel area, CsI scintillator directly deposited on amorphous Si photodiodes). For calibration of the experimental geometry, LaB_6_ SRM 660c NIST powder was prepared in a soda lime glass capillary with an internal diameter of 0.8 mm. The pyFAI software was used for the calibration of the experimental geometry and for azimuthal integration.^40^ The sample-to-detector distance (SDD) was calibrated to 300.613 mm. The area detector was placed such that the beam center was at the lower-right corner of the area detector when looking downstream from the sample position, resulting in a quarter of Debye–Scherrer rings collected on the detector. For the LaB_6_ SRM 660c NIST powder, total scattering data were collected for 300 s. The DUT-76(Cu) powder was prepared in a borosilicate capillary with an internal diameter of 0.5 mm, and total scattering data were collected for 1500 s. The background contribution to the total scattering data was collected for 1500 s using an empty borosilicate capillary with an internal diameter of 0.5 mm. To consider X-ray intensity fluctuations during data acquisition, the background data were scaled prior to subtraction from the DUT-76(Cu) data. To obtain the reduced atomic pair distribution function (PDF), the PDFgetX3 algorithm^41^ was used in the xPDFsuite software.^42^ The maximum value of momentum transfer for complete quarter rings on the area detector was *Q*_max,inst_ = 27.7 Å^–1^. However, to improve the signal-to-noise ratio of the resulting PDF, the Fourier transformation was terminated at *Q*_max_ = 17.9 Å^–1^, reflecting the poor counting statistics at higher Q-values due to limited signal of interest from the sample. The minimum value of momentum transfer included *Q*_min_ = 0.5 Å^–1^. For the ad hoc correction for incoherent scattering contribution, *r*_poly_ = 1.1, 1.12, or 1.13 Å was used.^41−43^

## Results and Discussion

### Standard Characterization of DUT-76(Cu)

**Figure 2:**
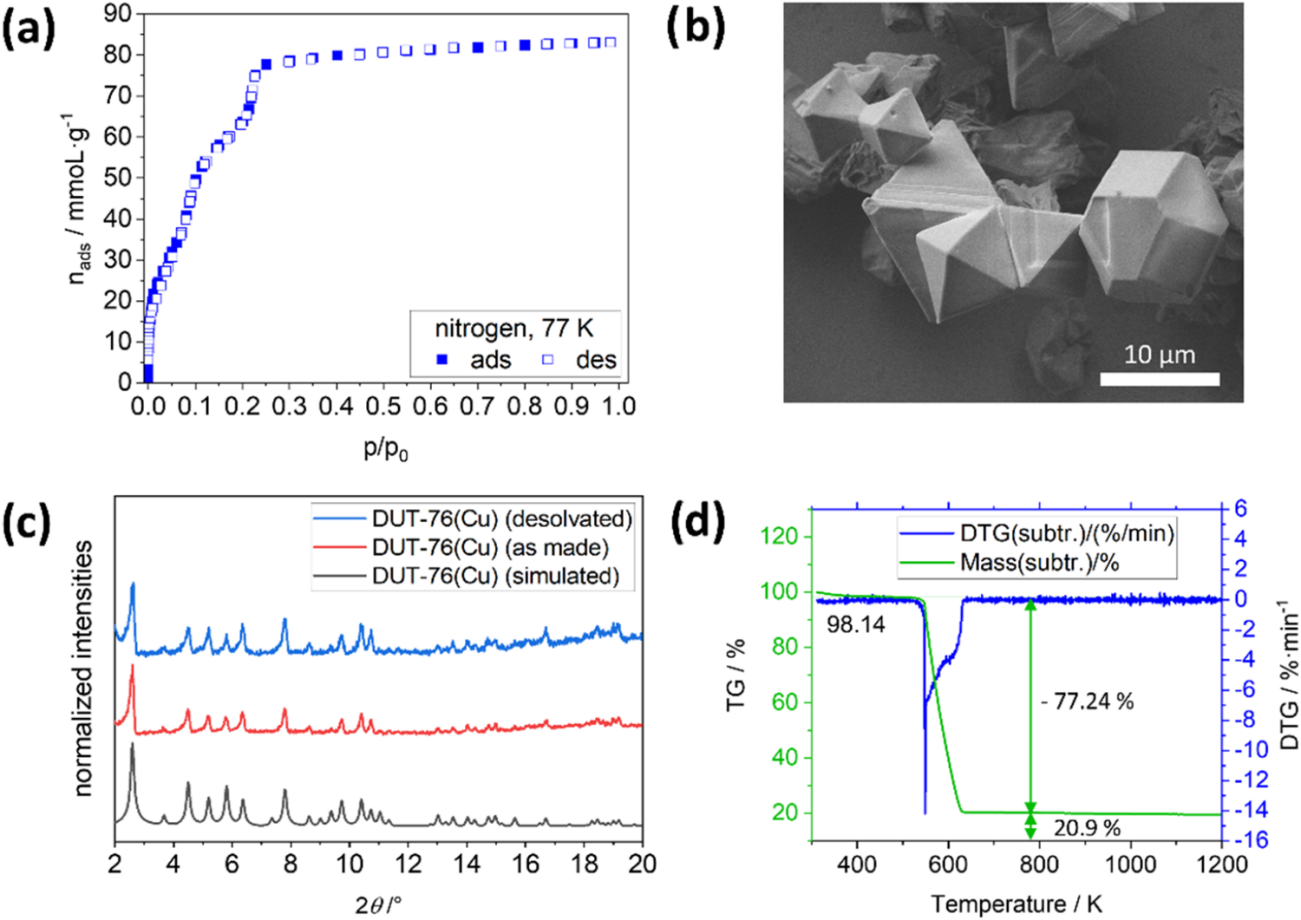
Characterization of DUT-76(Cu): (a) nitrogen isotherm at 77 K, (b) SEM image of desolvated crystals, (c) PXRD pattern of as-made and desolvated DUT-76(Cu) (λ = 0.15405 nm) with simulated pattern for comparison, and (d) thermal analysis in synthetic air.

The nitrogen isotherm measured for supercritically dried DUT-76(Cu) powder shows a type Ib isotherm (Figure 2) with three distinct steps before reaching a plateau at about 79.9 mmol·g^–1^(at *p*/*p*_0_ = 0.4). The steps in the isotherm are obviously related to the filling of three different cages (Figure 1) available in the crystal structure of the MOF: (1) the cuboctahedral pore with a diameter of 12 Å; (2) a truncated octahedral pore with a diameter of 21 Å; and (3) a cubic pore with a diameter of 27 Å.^34^ The TPV in saturation reached 2.77 cm^3^·g^–1^, which is in agreement with the calculated total geometric volume of 2.76 cm^3^·g^–1^ for this structure (see Figure 2a).^44^ SEM images of DUT-76(Cu) were obtained, indicating octahedral and cuboctahedral crystal shapes (see Figure 2b), and the PXRD patterns of the as-made and desolvated MOF were measured (see Figure 2c), and fit with the simulated pattern generated from the originally reported structure. The TGA graph shows a small step of 1.86% at 373 K due to moisture taken up while transferring the sample from a glovebox to the thermal analyzer. Then, from *T* = 533 to 638 K, a strong mass loss is observed due to the degradation of the linker, leaving 20.9% of the initial mass as copper(II) oxide, which is in agreement with the 21.9% calculated for this material.

### Investigation of the Interaction between DUT-76(Cu) and Different Hydrocarbons

**Figure 3:**
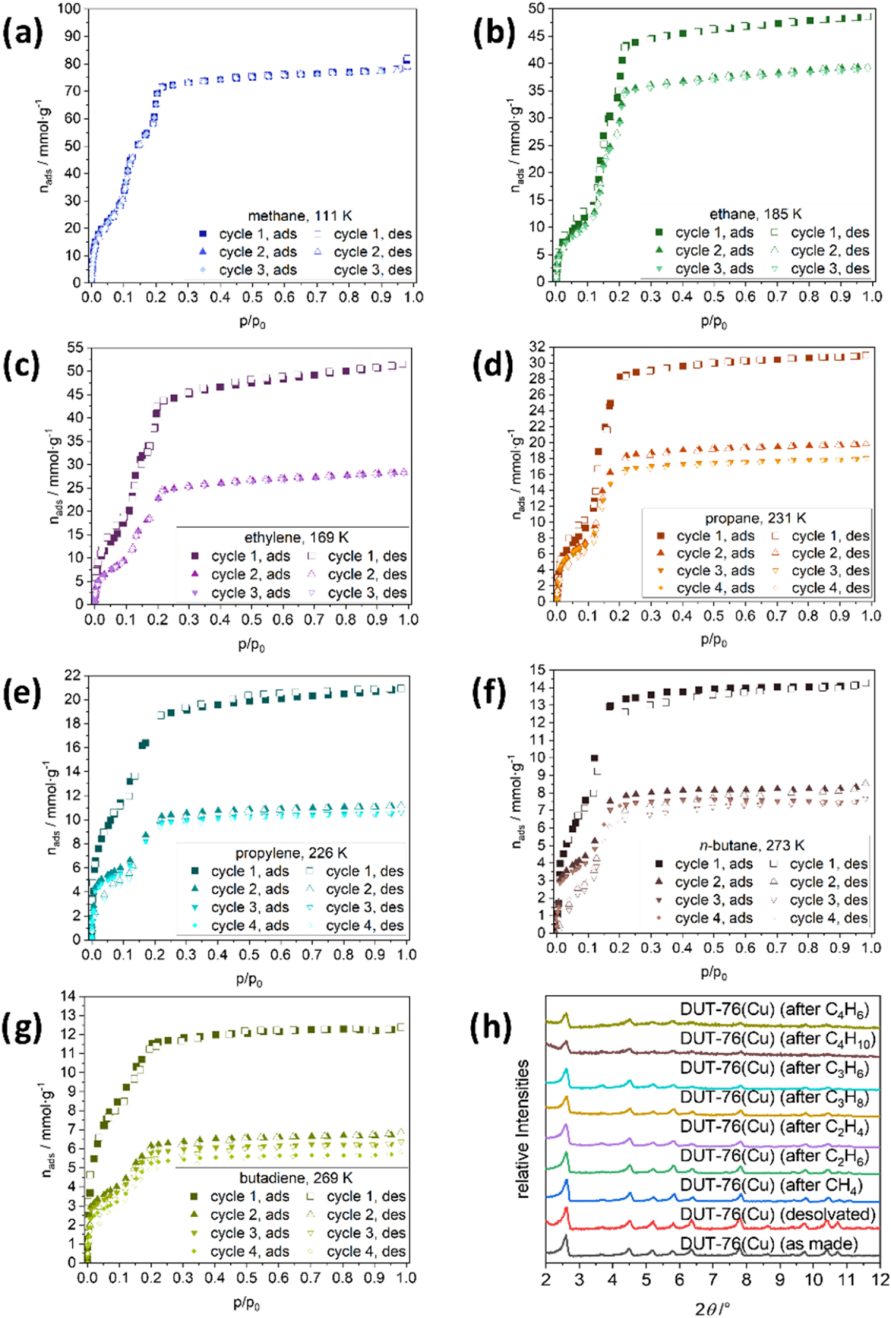
Adsorption isotherms of DUT-76(Cu) using various hydrocarbons at their respective boiling points, including multiple cycles: (a) methane at 111 K, (b) ethane at 185 K, (c) ethylene at 169 K, (d) propane at 231 K, (e) propylene at 226 K, (f) *n*-butane at 273 K, (g) 1,3-butadiene at 269 K, and (h) PXRD patterns of the samples after cycling experiments (λ = 0.15405 nm).

In order to prove the stability of DUT-76(Cu) toward adsorption/desorption stress, we conducted physisorption of C1–C4 hydrocarbons at their standard boiling points over several cycles (see Figure 3). In our previous work, we showed that the adsorption of hydrocarbons and noble gases close to their standard boiling points triggered breathing in the DUT-49(Cu) framework.^45^ Increasing the adsorption temperature suppressed the guest-induced flexibility in the pressure range of 0–1 bar, which was selected because of the broad availability of low-pressure adsorption devices. Moreover, the adsorption of *n*-butane at 273 K can be easily conducted using basic equipment for physisorption measurements.

We started our experiments with methane physisorption at 111 K because methane shows the weakest interactions with the framework among all hydrocarbons. The isotherm shape upon methane physisorption is very similar to that of nitrogen, with two steps until *p*/*p*_0_ = 0.2, and then reaches a plateau at *p*/*p*_0_ = 0.3 (see Figure 3a). The total uptake of methane in the plateau is 74.3 mmol·g^–1^ (at *p*/*p*_0_ = 0.4), corresponding to TPV of 2.81 cm^3^·g^–1^. The TPV values of DUT-76(Cu) differ depending on the adsorbed hydrocarbons. An in-depth discussion is provided below (see Figure 6). The physisorption is repeatable over at least three cycles without changes, although a narrow hysteresis is observed in the pressure range *p*/*p*_0_ = 0–0.1, corresponding to adsorption and desorption of methane in the cuboctahedral pore, obviously without deteriorating the crystal structure.

As DUT-76(Cu) showed exceptional ethene uptake in high-pressure adsorption experiments at 298 K, we conducted an in-depth fundamental study of host–guest interactions with hydrocarbons of different lengths and degrees of saturation at their boiling points. When comparing the isotherms of ethane and ethylene, a significant influence of the degree of saturation was discerned (see Figure 3b,c). An almost identical uptake of ethane (45.5 mmol·g^–1^, TPV = 2.52 cm^3^·g^–1^, at *p*/*p*_0_ = 0.40, 185 K) and ethylene (46.6 mmol·g^–1^, TPV = 2.30 cm^3^·g^–1^, at *p*/*p*_0_ = 0.40, 169 K) was reached in the first cycle. This is 0.3 and 0.5 cm^3^g^–1^ lower than that of methane, indicating that 10 and 18% of the DUT-76(Cu) sample, respectively, irreversibly contracted during exposure to adsorption stress. However, the three steps in the isotherm observed for nitrogen and methane are still visible. For both gases, we observed two narrow hysteretic loops in the isotherms. The first one, similar to methane, appears in the first step, and the second one is observed between the second and third steps in the range of *p*/*p*_0_ = 0.1–0.15. In the second physisorption cycle, however, the uptake of the two gases decreased significantly. Namely, for ethane, a drop in gas uptake of about 20% to 36.8 mmol·g^–1^ (at *p*/*p*_0_ = 0.40, 185 K) is observed, whereas, for ethylene, this drop is more significant as the maximum uptake decreased to almost 50% to 26.1 mmol·g^–1^ (at *p*/*p*_0_ = 0.40, 169 K). The changes are obviously related to the desorption of gas from the mesopores in the first adsorption/desorption cycle, indicating that the framework is exposed to a larger stress upon desorption of ethylene. The second- and third-cycle isotherms show reversible behavior with no hysteresis. This observation, as well as the fact that the sample was heated to 298 K and evacuated between measurements, also shows that the lower uptake is not due to the adsorbate remaining in the pores but to changes in the framework itself.

**Figure 4:**
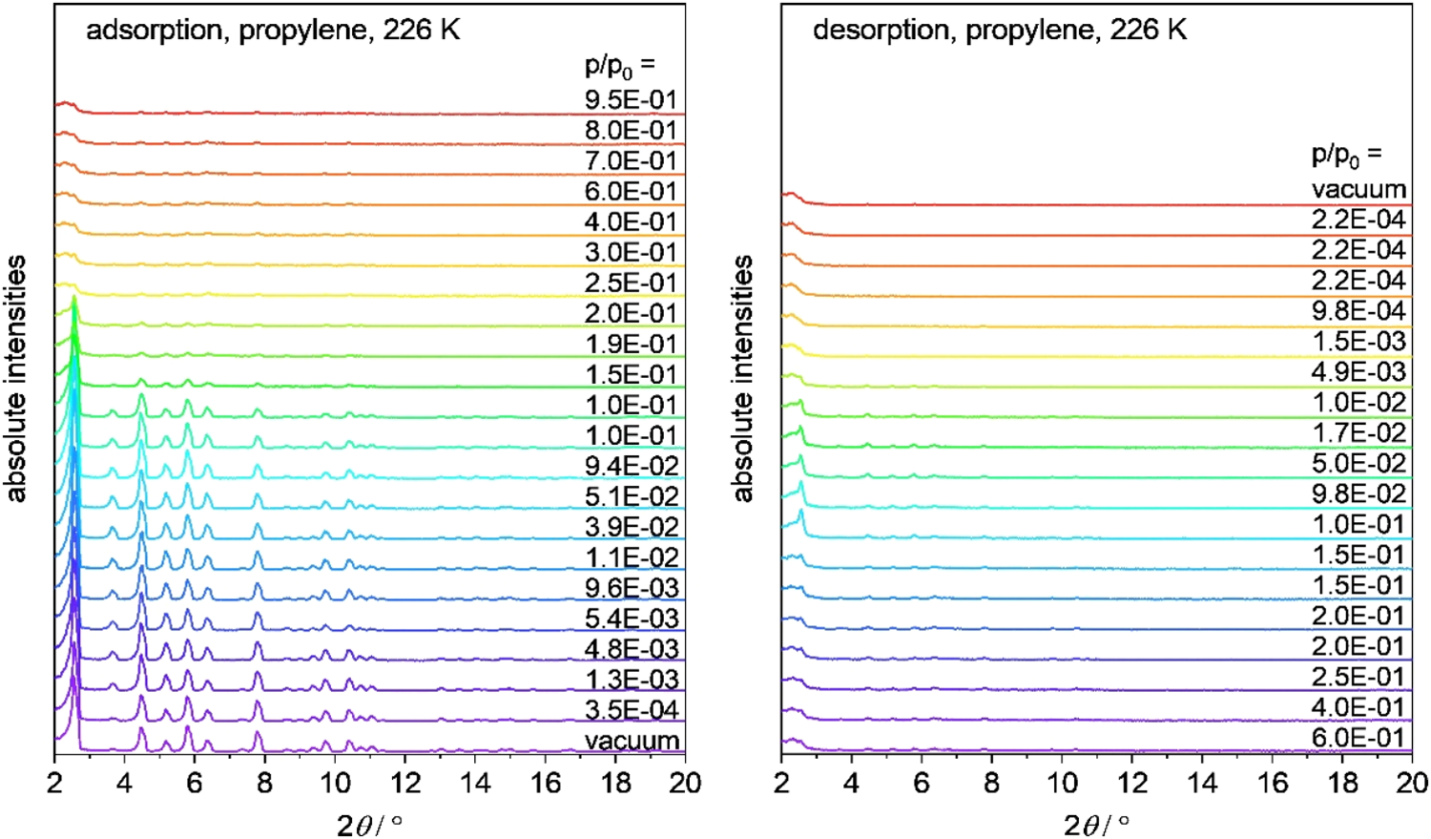
*In situ* PXRD patterns (λ = 0.15405 nm) along with the adsorption (left) and desorption (right) of propylene at 226 K.

**Figure 5:**
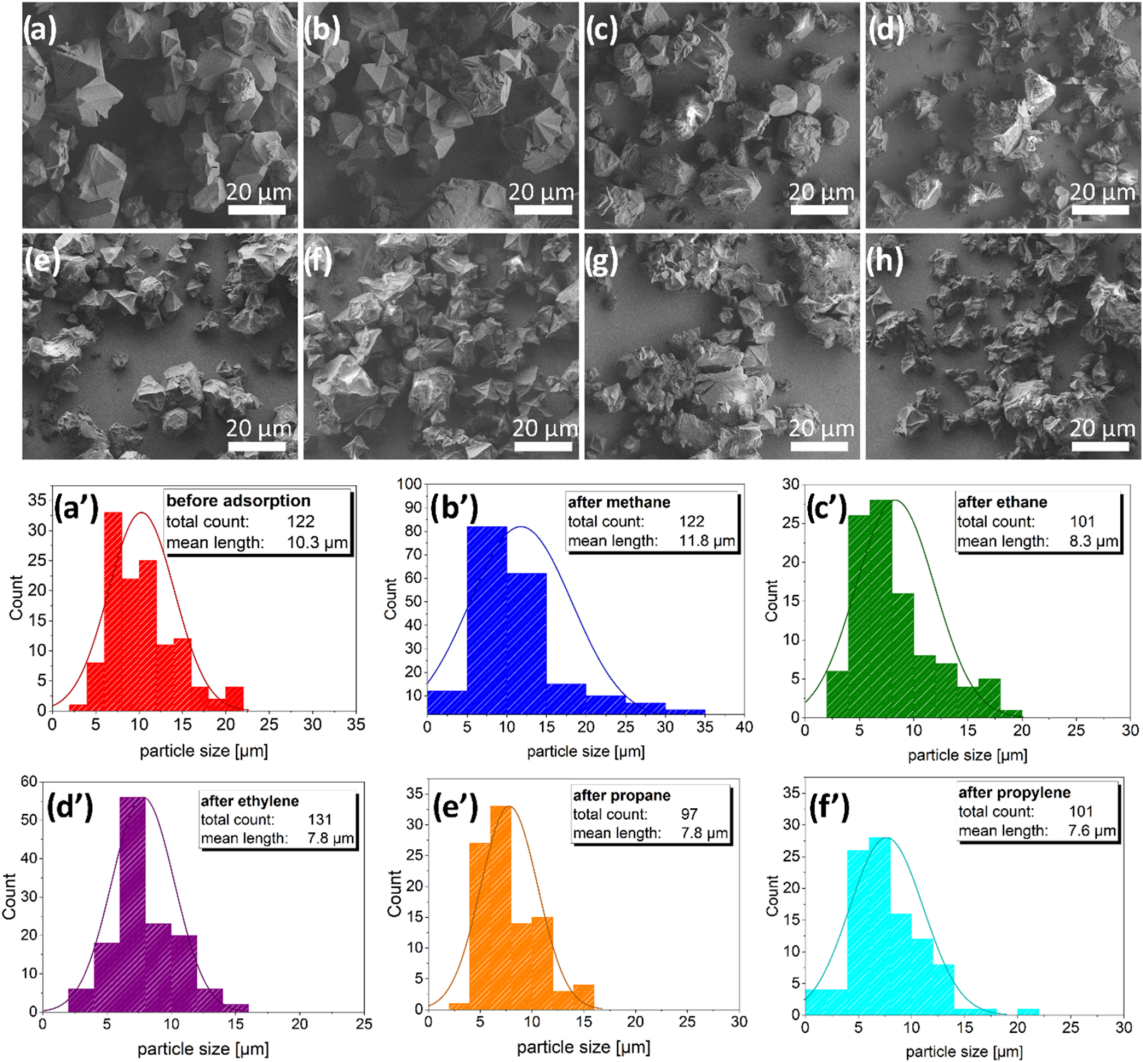
SEM images of the DUT-76(Cu) crystals. (a) Before physisorption experiment and after 1 cycle of adsorption/desorption of (b) methane, (c) ethane, (d) ethylene, (e) propane, (f) propylene, (g) *n-*butane, (h) 1,3-butadiene, and (a′–f′) corresponding particle size distributions.

The physisorption of propylene and propane shows a trend similar to that observed for ethylene and ethane. A higher uptake of propane (29.6 mmol·g^–1^, TPV = 2.30 cm^3^·g^–1^, at *p*/*p*_0_ = 0.40, 231 K) than that of propylene (19.6 mmol·g^–1^, TPV = 1.35 cm^3^·g^–1^, at *p*/*p*_0_ = 0.40, 226 K) is observed (see Figure 3d,e), indicating that the latter exposes the framework to higher adsorption stress, resulting in irreversible contraction of 18 and 52% in the MOF, respectively. Interestingly, the third step of the isotherm is almost eliminated, either by increasing the molecular size of the guest or by contraction of the mesopores in the first adsorption cycle. The first cycle of the propane isotherm shows a narrow hysteresis at a relative pressure of *p*/*p*_0_ = 0.0–0.2; however, we can also see the desorption branch is positioned underneath the adsorption branch, indicating that desorption occurs at a higher relative pressure than the adsorption. This can be explained by a partial decomposition of the material upon guest adsorption, followed by an earlier gas release from this partially decomposed phase. This effect of the material’s decomposition as well as its premature gas release became stronger as host–guest interactions increased, and it was investigated using *in situ* PXRD (Figures 4, S6, S8, and S10) as well as SEM imaging (see Figure 5). In the following cycles, the uptake drops to 64% and then further to 6%, and then remains almost constant in the fourth cycle. For propylene, the behavior is almost identical but without hysteresis in the first adsorption/desorption step. The uptake drops to about 55% in the second cycle and slightly decreases to 52% in the third and fourth cycles. For both gases, we observed the crossing of the adsorption and desorption branches within the second step of the isotherm, indicating partial decomposition of the sample.

DUT-76(Cu) takes up more of the saturated hydrocarbon *n*-butane (13.7 mmol·g^–1^, TPV = 1.33 cm^3^·g^–1^, at *p*/*p*_0_ = 0.40, 273 K), followed by a drop to 60% in the second cycle and further 5% in the third cycle, where the uptake then remains constant. The lowest uptake was observed for unsaturated 1,3-butadiene (12.0 mmol·g^–1^, TPV = 0.99 cm^3^·g^–1^, at *p*/*p*_0_ = 0.40, 269 K), followed by a drop to 55% and then further decrease in the uptake with 5% in each step (see Figure 3f,g).

Partial irreversible contraction of the framework during adsorption/desorption cycling can also be observed in *ex situ* PXRD experiments conducted on the samples before and after the cycling experiments (see Figure 3h). The reflection intensities gradually decrease with increasing chain length of the hydrocarbons, used in the adsorption/desorption cycling experiments, although part of the sample still retains its crystallinity. This observation is in good agreement with the physisorption experiments, indicating reduced gas uptake and the pristine shape of the isotherm.

### *In Situ* PXRD Experiments in Parallel to Gas Adsorption

As the gas adsorption isotherms and *ex situ* PXRD patterns indicate the changes in the material, *in situ* PXRD measurements in parallel with the adsorption of various gases should provide a clear analysis of the structural transitions occurring during gas adsorption. The results are in agreement with the previous observation, as the reflections show the characteristic pattern of DUT-76(Cu) but the intensity rapidly decreases in the pressure range *p*/*p*_0_ = 0.1–0.19, which is when the smallest pores – the cuboctahedral MOPs – are filled and the larger tetragonal and ultimately the cubic pores fill up (see Figure 4). In the desorption branch, a further decrease in the reflection intensity is observed. Subtle changes in the diffraction pattern are visible, namely a transient increase of the peak at 2θ = 3.7° from *p*/*p*_0_ = 0.0–0.039. In the higher angle range of 2θ > 8.0°, the reflection intensity drops and then increases again with a minimum at *p*/*p*_0_ = 0.0054. However, the formation of new crystalline phases was not observed. *In situ* PXRD, measured in parallel with the physisorption of propane and *n-*butane, shows comparable results (see Figures S2–S6). The decrease in the reflection intensity can be attributed to either decomposition or amorphization. Thus, the material remains porous (see Figure 3) and the local order remains intact (see Figure 7). We attribute the change in the reflection intensities mainly to amorphization.

### Crystal Morphology by SEM Imaging

Since we observed partial decomposition of the samples upon physisorption of C2–C4 hydrocarbons and no new crystalline phases appeared in the PXRD patterns, SEM imaging was applied to analyze the changes on the macroscopic scale (see Figure 5).

The crystals exhibited different degrees of deformation. The crystals used for the adsorption/desorption experiments had octahedral or truncated octahedral shapes (Figure 5a), but some broken crystals were already visible. The size distribution was broad (Figure 5a′) but most crystals were between 5 and 15 μm in diameter. After methane adsorption at 111 K, the crystals did not change the shape and morphology; however, the average crystal size decreased slightly (Figure 5b,b′). In the case of ethane (Figure 5c,c′) and ethene (Figure 5d,d′) physisorption, a significant number of crystals exhibited concave, wrinkled surfaces, and cracks, and smaller particles were observed in the sample after physisorption. The same trend was observed for propane (Figure 5e,e′) and propylene (Figure 5f,f′), in which the crystal shape could still be estimated, but the crystal underwent significant degradation. In the case of *n*-butane (Figure 5g) and 1,3-butadiene (Figure 5h), almost the entire initial habitus vanished, and mainly fragments or strongly decomposed material were visible. These observations are in line with the physisorption and PXRD results, indicating the contraction of the MOF with an increase in the guest size. The decrease in the size of the intact crystals and the amount of fragmented larger crystals can be explained by the stabilization of smaller crystals due to a smaller ratio of inner and outer surfaces.^22^

### Total Pore Volume Evolution as a Function of Adsorption/Desorption Stress

Hydrocarbon physisorption showed a gradual decrease in gas adsorption as the chain length of the adsorbate increased and the double bonds were introduced. Both effects are likely to increase the amount of adsorption/desorption stress on the framework: longer chains lead to an increase of van der Waals (VdW) forces, and the presence of double bonds introduces the possibility of π–π interactions. Both factors lead to an increase in the adsorption enthalpy and adsorption stress, which leads to framework contraction.

**Figure 6:**
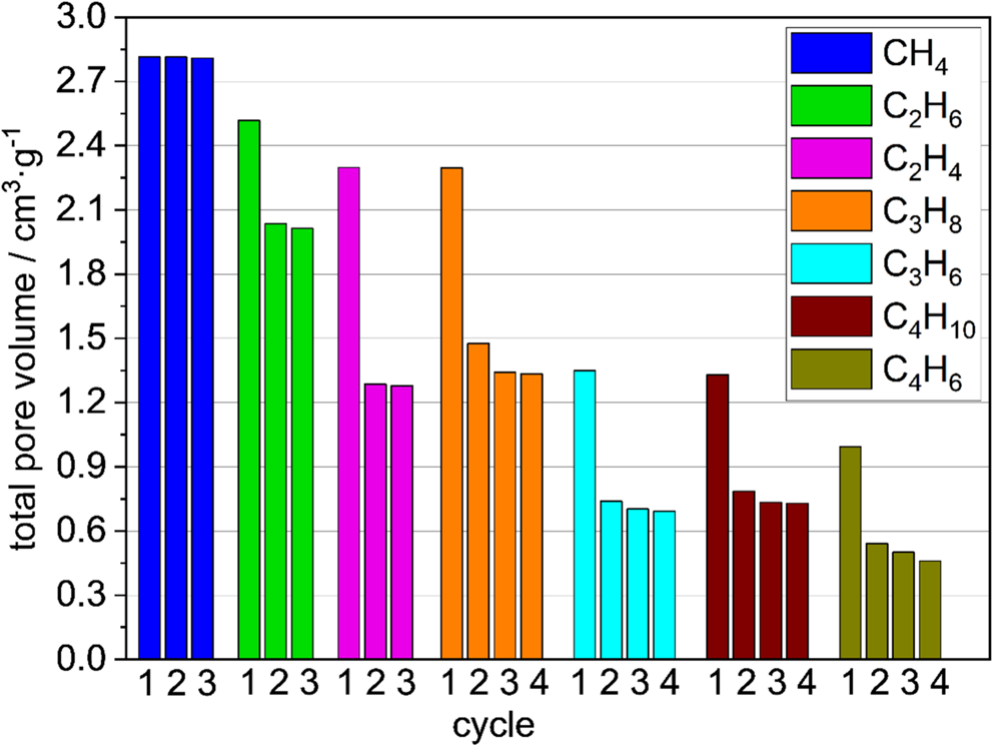
Comparison of TPV of DUT-76(Cu) with different gases at their standard boiling points and multiple cycles. The TPV was calculated using the isotherms described above.

The underlying phenomena and correlations were clearly discerned by plotting the total pore volumes (Figure 6) determined in multiple cycles with C1–C4 alkanes and alkenes. For methane, we observed the highest pore volume of 2.81 cm^3^·g^–1^, which was also observed in the nitrogen physisorption measurements. This value does not change significantly over the three adsorption/desorption cycles. For methane, the energy input through the adsorption enthalpy in adsorption and capillary forces in desorption is insufficient to contract the structure, leading to reproducible and repeatable adsorption over multiple cycles. This is reflected in the SEM images, as the majority of the crystals remain intact, their faces remain plain, and their geometric shape and surface do not change after multiple cycles.

For C2 hydrocarbons, a lower TPV is observed, indicating the partial contraction of the sample during adsorption. Following the trends for the rigidification of flexible MOFs, a reasonable hypothesis is that larger crystals collapse completely, while smaller crystals resist the contractive adsorption stress and maintain the pore volume of the sample. For ethane, the TPV was decreased to 2.52 cm^3^·g^–1^ and then further decreased by 20% to 2.01 cm^3^·g^–1^ after the third cycle, indicating a transition into a less porous structure. For ethylene, the TPV was further decreased to 2.30 cm^3^·g^–1^ and after three cycles, it decreased by 44% to 1.28 cm^3^·g^–1^. This result is in agreement with other findings, indicating a higher isosteric heat of adsorption with increasing carbon chain length due to stronger van der Waals interactions and additional π–π interactions when double bonds are introduced but the chain length remains the same.^46,47^ This lower TPV also correlates with the mean crystal size decreasing from 8.3 to 7.8 μm as well as the visible changes in the crystal morphology (Figure 5).

In transitioning from C2 to C3 hydrocarbons, the TPV for propane is 2.30 cm^3^·g^–1^ and decreased by 42% to 1.33 cm^3^·g^–1^, which is very similar to the value for ethylene. A fourth adsorption/desorption cycle was performed; thus, the pore volume in the third cycle differed significantly from that in the second cycle. For propylene, the TPV in the first cycle was 1.35 cm^3^·g^–1^ with a 49% decrease to 0.69 cm^3^·g^–1^ after four cycles. Again, these values were very similar to those for *n*-butane, where a TPV of 1.33 cm^3^·g^–1^ with a 45% decrease to 0.73 cm^3^·g^–1^ was observed. The similarity in TPV for the ethylene/propane and propylene/*n*-butane pairs is noticeable and may be explained by increasing van der Waals interactions, compensating for the lack of π–π interactions and vice versa. For 1,3-butadiene, we observed the lowest total pore volume of 0.99 cm^3^·g^–1^ and a decrease to 0.46 cm^3^·g^–1^ after four cycles.

These results correlate well, the adsorption stress from different alkanes larger than methane and alkenes causes not only an irreversible transition into an amorphous phase but also macroscopic deformation and fragmentation of the crystals when the adsorption/desorption stress becomes too strong. This leads to a decrease in the gas uptake and total pore volume. An interesting empirical observation could be derived from the physisorption data, that is, the adsorption of the alkene with *n* carbon atoms in the chain exposes the framework to the adsorption/desorption stress, similar to the alkane with *n+1* carbon atoms in the molecule, indicating a significant contribution of *π-π* interactions. Qualitatively, the adsorption stress increases with increasing boiling point and adsorption enthalpy of the probe molecule, reflecting increasing dispersive interactions. Since the framework topology does not allow the transformation into another crystalline contracted phase, irreversible contraction (collapse) was observed.

### Total Scattering Experiments with DUT-76(Cu)

**Figure 7:**
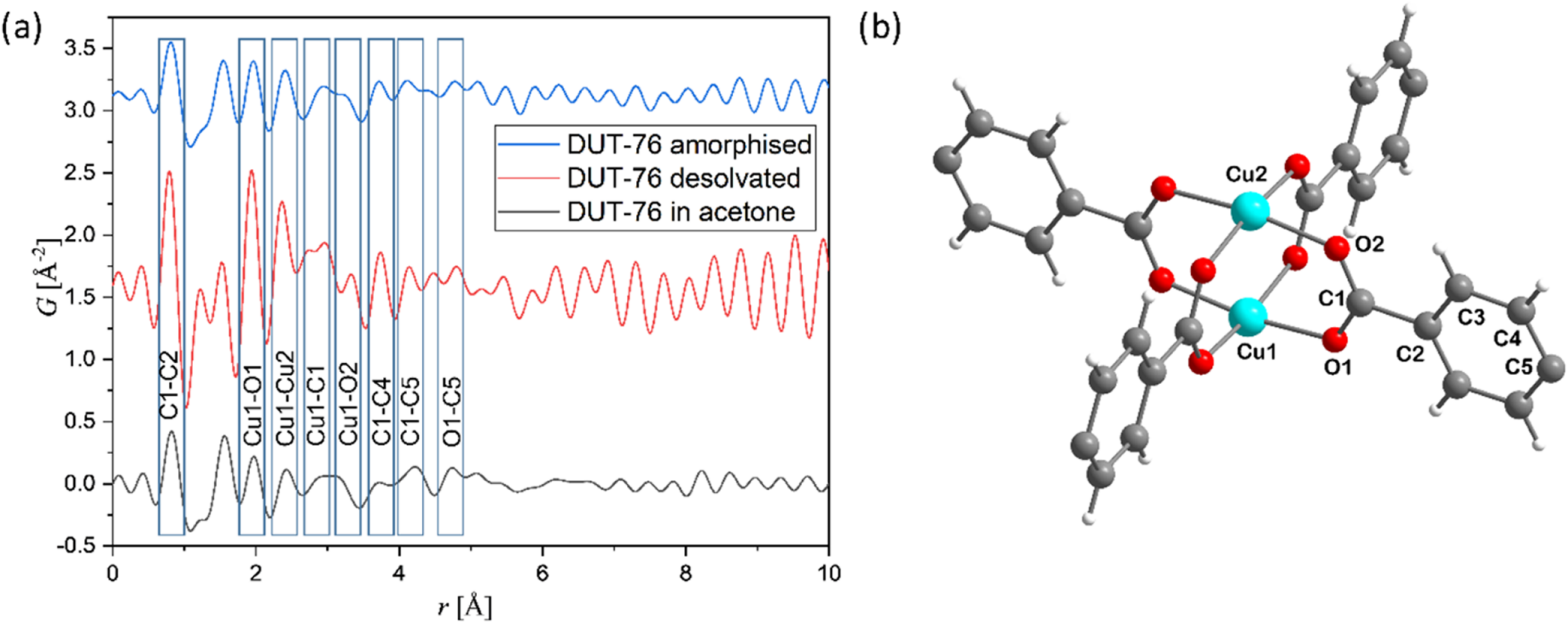
(a) Fourier-transformed PDF, plotted for DUT-76(Cu) immersed in acetone, desolvated, and amorphized contracted samples, and (b) local structure of the copper dimeric cluster in DUT-76(Cu).

In order to elucidate the local structure of the contracted amorphous phase, total scattering experiments were conducted on DUT-76(Cu) samples (immersed in acetone, desolvated using supercritical CO_2_ drying, and completely amorphized upon aceton desorption at 298 K) at the P02.1 beamline of the PETRA III synchrotron. The analysis of Fourier-transformed PDF indicates only minor changes in the coordination geometry of copper, showing only minor shifts in the Cu1–O1 and Cu1–Cu2 distances toward smaller values in the desolvated framework (see Figure 7). In addition, the typical carbon–carbon and carbon–oxygen distances in the phenyl ring are in the range of 1–5 Å. Interestingly, the distances corresponding to the peaks in the range between 1.5 and 2.0 Å could not be located in the crystal structure of DUT-76. The local structure of the crystalline framework, immersed in acetone, and the amorphized contracted structure are very similar in the distance range below 4 Å, indicating that only long-range order and porosity change after contraction. Hence, the framework collapse largely retains the local structure of the framework, but pore collapse causes a loss of long-range order and framework deformation.

## Conclusions

In summary, we employed DUT-76(Cu) as a model system to evaluate the mechanical stability of mesoporous frameworks using experimental adsorption techniques based on the gradual increase in the adsorption/desorption stress over multiple cycles of physisorption with C1–C4 hydrocarbons at their standard boiling points. The results indicate partial contraction of the sample with increasing chain length or introduction of double bonds. Our observations reveal a growing fraction of the collapsed frameworks with increasing probe molecule chain length, the presence of double bonds, and the number of adsorption/desorption cycles. For all gases, stable adsorption–desorption performance was achieved after the third cycle.

A combination of gas physisorption, X-ray diffraction, SEM imaging, and total scattering enabled quantification of the contracted fraction of the sample, monitoring of changes in crystallinity and macroscopic crystal morphology, and confirmation of the local structure of both crystalline and amorphized phases. The proposed technique provides a semiquantitative approach for evaluating the mechanical stability of highly sensitive mesoporous frameworks and serves as a potential alternative to existing methods.

## Supporting Information Available

Material purity and suppliers; detailed experimental procedures for the synthesis of the linker with ^1^H and ^13^C nuclear magnetic resonance (NMR) spectroscopy and high-resolution mass spectrometry (HRMS) data; in-depth description of the solvothermal synthesis and desolvation of DUT-76(Cu) powder; photographs of activated and decomposed DUT-76(Cu) powder; and isotherms and PXRD patterns of *in situ* measurements in parallel to the adsorption of propane, propylene, and *n*-butane. (PDF)
Additional data. (ZIP)

## References

[ref1] Hönicke I. M.; Senkovska I.; Bon V.; Baburin I. A.; Bönisch N.; Raschke S.; Evans J. D.; Kaskel S. Balancing Mechanical Stability and Ultrahigh Porosity in Crystalline Framework Materials. Angew. Chem., Int. Ed. 2018, 57 (42), 13780–13783. 10.1002/anie.201808240. 30160076

[ref2] Furukawa H.; Ko N.; Go Y. B.; Aratani N.; Choi S. B.; Choi E.; Yazaydin A. Ö.; Snurr R. Q.; O’Keeffe M.; Kim J.; Yaghi O. M.; O’Keeffe M.; Kim J.; Yaghi O. M. Ultrahigh Porosity in Metal-Organic Frameworks. Science 2010, 329 (5990), 424–428. 10.1126/science.1192160. 20595583

[ref3] Farha O. K.; Eryazici I.; Jeong N. C.; Hauser B. G.; Wilmer C. E.; Sarjeant A. A.; Snurr R. Q.; Nguyen S. T.; Yazaydın A. Ö.; Hupp J. T. Metal-Organic Framework Materials with Ultrahigh Surface Areas: Is the Sky the Limit?. J. Am. Chem. Soc. 2012, 134 (36), 15016–15021. 10.1021/ja3055639. 22906112

[ref4] Krause S.; Bon V.; Senkovska I.; Stoeck U.; Wallacher D.; Többens D. M.; Zander S.; Pillai R. S.; Maurin G.; Coudert F.-X.; Kaskel S. A pressure-amplifying framework material with negative gas adsorption transitions. Nature 2016, 532 (7599), 348–352. 10.1038/nature17430. 27049950

[ref5] Krause S.; Hosono N.; Kitagawa S. Chemistry of Soft Porous Crystals: Structural Dynamics and Gas Adsorption Properties. Angew. Chem., Int. Ed. 2020, 59 (36), 15325–15341. 10.1002/anie.202004535. 32458545

[ref6] Tian Y.-J.; Deng C.; Peng Y.-L.; Zhang X.; Zhang Z.; Zaworotko M. J. State of the art, challenges and pprospects in metal-organic frameworks for the separation of binary propylene/propane mixtures. Coord. Chem. Rev. 2024, 506, 215697 10.1016/j.ccr.2024.215697.

[ref7] Park J.; Adhikary A.; Moon H. R. Progress in the development of flexible metal-organic frameworks for hydrogen storage and selective separation of its isotopes. Coord. Chem. Rev. 2023, 497, 215402 10.1016/j.ccr.2023.215402.

[ref8] Senkovska I.; Bon V.; Abylgazina L.; Mendt M.; Berger J.; Kieslich G.; Petkov P.; Luiz Fiorio J.; Joswig J.; Heine T.; Schaper L.; Bachetzky C.; Schmid R.; Fischer R. A.; Pöppl A.; Brunner E.; Kaskel S. Understanding MOF Flexibility: An Analysis Focused on Pillared Layer MOFs as a Model System. Angew. Chem., Int. Ed. 2023, 62, e202218076 10.1002/anie.202218076. 37052183

[ref9] Bon V.; Brunner E.; Pöppl A.; Kaskel S. Unraveling Structure and Dynamics in Porous Frameworks via Advanced In Situ Characterization Techniques. Adv. Funct. Mater. 2020, 30, 1907847 10.1002/adfm.201907847.

[ref10] Zhang J.-P.; Zhou H.-L.; Zhou D.-D.; Liao P.-Q.; Chen X.-M. Controlling flexibility of metal-organic frameworks. Natl. Sci. Rev. 2018, 5 (6), 907–919. 10.1093/nsr/nwx127.

[ref11] Krause S.; Bon V.; Senkovska I.; Többens D. M.; Wallacher D.; Pillai R. S.; Maurin G.; Kaskel S. The effect of crystallite size on pressure amplification in switchable porous solids. Nat. Commun. 2018, 9, 1573 10.1038/s41467-018-03979-2. 29679030 PMC5910435

[ref12] Wang J.; Imaz I.; Maspoch D. Metal-Organic Frameworks: Why Make Them Small?. Small Struct. 2022, 3, 2100126 10.1002/sstr.202270002.

[ref13] Zhang C.; Gee J. A.; Sholl D. S.; Lively R. P. Crystal-Size-Dependent Structural Transitions in Nanoporous Crystals: Adsorption-Induced Transitions in ZIF-8. J. Phys. Chem. C 2014, 118, 20727–20733. 10.1021/jp5081466.

[ref14] Vandenhaute S.; Rogge S. M. J.; Van Speybroeck V. Large-Scale Molecular Dynamics Simulations Reveal New Insights Into the Phase Transition Mechanisms in MIL-53(Al). Front. Chem. 2021, 9, 718920 10.3389/fchem.2021.718920. 34513797 PMC8429608

[ref15] Abylgazina L.; Senkovska I.; Engemann R.; Bönisch N.; Gorelik T. E.; Bachetzky C.; Kaiser U.; Brunner E.; Kaskel S. Chemoselectivity Inversion of Responsive Metal-Organic Frameworks by Particle Size Tuning in the Micrometer Regime. Small 2024, 20, 2307285 10.1002/smll.202307285. 38225688

[ref16] Tanaka S.; Fujita K.; Miyake Y.; Miyamoto M.; Hasegawa Y.; Makino T.; Van der Perre S.; Cousin Saint Remi J.; Van Assche T.; Baron G. V.; Denayer J. F. M. Adsorption and Diffusion Phenomena in Crystal Size Engineered ZIF-8 MOF. J. Phys. Chem. C 2015, 119 (51), 28430–28439. 10.1021/acs.jpcc.5b09520.

[ref17] Johnson L. J. W.; Mirani D.; Le Donne A.; Bartolomé L.; Amayuelas E.; López G. A.; Grancini G.; Carter M.; Yakovenko A. A.; Trump B. A.; Meloni S.; Zajdel P.; Grosu Y. Effect of Crystallite Size on the Flexibility and Negative Compressibility of Hydrophobic Metal-Organic Frameworks. Nano Lett. 2023, 23, 10682–10686. 10.1021/acs.nanolett.3c02431. 38033298 PMC10722533

[ref18] Krause S.; Bon V.; Du H.; Dunin-Borkowski R. E.; Stoeck U.; Senkovska I.; Kaskel S. The impact of crystal size and temperature on the adsorption-induced flexibility of the Zr-based metal-organic framework DUT-98. Beilstein J. Nanotechnol. 2019, 10, 1737–1744. 10.3762/bjnano.10.169. 31501745 PMC6719731

[ref19] Bon V.; Busov N.; Senkovska I.; Bönisch N.; Abylgazina L.; Khadiev A.; Novikov D.; Kaskel S. The importance of crystal size for breathing kinetics in MIL-53(Al). Chem. Commun. 2022, 58 (75), 10492–10495. 10.1039/D2CC02662G. 36043355

[ref20] Ma Y.; Yu C.; Yang L.; You R.; Bo Y.; Gong Q.; Xing H.; Cui X. Boosting kinetic separation of ethylene and ethane on microporous materials via crystal size control. Chin. J. Chem. Eng. 2024, 65, 85–91. 10.1016/j.cjche.2023.06.001.

[ref21] Ehrling S.; Miura H.; Senkovska I.; Kaskel S. From Macro- to Nanoscale: Finite Size Effects on Metal-Organic Framework Switchability. Trends Chem. 2021, 3 (4), 291–304. 10.1016/j.trechm.2020.12.012.

[ref22] Bon V.; Kavoosi N.; Senkovska I.; Kaskel S. Tolerance of flexible MOFs towards repeated adsorption stress. ACS Appl. Mater. Interfaces 2015, 7 (40), 22292–22300. 10.1021/acsami.5b05456. 26397165

[ref23] Coudert F. X.; Boutin A.; Fuchs A. H.; Neimark A. V. Adsorption Deformation and Structural Transitions in Metal-Organic Frameworks: From the Unit Cell to the Crystal. J. Phys. Chem. Lett. 2013, 4 (19), 3198–3205. 10.1021/jz4013849. 23163384

[ref24] Kolesnikov A. L.; Budkov Y. A.; Gor G. Y. Models of adsorption-induced deformation: ordered materials and beyond. J. Phys.: Condens. Matter 2022, 34 (6), 063002 10.1088/1361-648X/ac3101. 34666316

[ref25] Meng Q.; Wang L.; Yang D.; Xing J. Methane and helium adsorption of coal and its related deformation under different temperature and pressures. Energy Sources, Part A 2022, 44 (2), 3929–3944. 10.1080/15567036.2022.2072025.

[ref26] Kowalczyk P.; Ciach A.; Neimark A. V. Adsorption-Induced Deformation of Microporous Carbons: Pore Size Distribution Effect. Langmuir 2008, 24, 6603–6608. 10.1021/la800406c. 18522449

[ref27] Gor G. Y.; Neimark A. V. Adsorption-Induced Deformation of Mesoporous Solids: Macroscopic Approach and Density Functional Theory. Langmuir 2011, 27, 6926–6931. 10.1021/la201271p. 21568283

[ref28] Neimark A. V.; Coudert F.-X.; Boutin A.; Fuchs A. H. Stress-Based Model for the Breathing of Metal–Organic Frameworks. J. Phys. Chem. Lett. 2010, 1, 445–449. 10.1021/jz9003087. 26700628

[ref29] Yot P. G.; Ma Q.; Haines J.; Yang Q.; Ghoufi A.; Devic T.; Serre C.; Dmitriev V.; Férey G.; Zhong C.; Maurin G. Large breathing of the MOF MIL-47(V^IV^) under mechanical pressure: a joint experimental–modelling exploration. Chem. Sci. 2012, 3, 1100–1104. 10.1039/c2sc00745b.

[ref30] Yot P. G.; Vanduyfhuys L.; Alvarez E.; Rodriguez J.; Itié J.-P.; Fabry P.; Guillou N.; Devic T.; Beurroies I.; Llewellyn P. L.; Van Speybroeck V.; Serre C.; Maurin G. Mechanical energy storage performance of an aluminum fumarate metal-organic framework. Chem. Sci. 2016, 7, 446–450. 10.1039/C5SC02794B. 29861993 PMC5952545

[ref31] Redfern L. R.; Farha O. K. Mechanical properties of metal-organic frameworks. Chem. Sci. 2019, 10, 10666–10679. 10.1039/C9SC04249K. 32190239 PMC7066669

[ref32] Rogge S. M. J.; Yot P. G.; Jacobsen J.; Muniz-Miranda F.; Vandenbrande S.; Gosch J.; Ortiz V.; Collings I. E.; Devautour-Vinot S.; Maurin G.; Stock N.; Van Speybroeck V. Charting the Metal-Dependent High-Pressure Stability of Bimetallic UiO-66 Materials. ACS Mater. Lett. 2020, 2, 438–445. 10.1021/acsmaterialslett.0c00042. 32296781 PMC7147928

[ref33] Moghadam P. Z.; Rogge S. M. J.; Li A.; Chow C.-M.; Wieme J.; Moharrami N.; Aragones-Anglada M.; Conduit G.; Gomez-Gualdron D. A.; Van Speybroeck V.; Fairen-Jimenez D. Structure-Mechanical Stability Relations of Metal-Organic Frameworks via Machine Learning. Matter 2019, 1, 219–234. 10.1016/j.matt.2019.03.002.

[ref34] Stoeck U.; Senkovska I.; Bon V.; Krause S.; Kaskel S. Assembly of metal-organic polyhedra into highly porous frameworks for ethene delivery. Chem. Commun. 2015, 51, 1046–1049. 10.1039/C4CC07920E. 25434790

[ref35] Krause S.; Evans J. D.; Bon V.; Senkovska I.; Iacomi P.; Kolbe F.; Ehrling S.; Troschke E.; Getzschmann J.; Többens D. M.; Franz A.; Wallacher D.; Yot P. G.; Maurin G.; Brunner E.; Llewellyn P. L.; Coudert F.-X.; Kaskel S. Towards general network architecture design criteria for negative gas adsorption transitions in ultraporous frameworks. Nat. Commun. 2019, 10, 3632 10.1038/s41467-019-11565-3. 31406113 PMC6690989

[ref36] Walenszus F.; Evans J. D.; Bon V.; Schwotzer F.; Senkovska I.; Kaskel S. Integration of Fluorescent Functionality into Pressure-Amplifying Metal-Organic Frameworks. Chem. Mater. 2021, 33, 7964–7971. 10.1021/acs.chemmater.1c01804. 35600608 PMC9115756

[ref37] Krause S.; Evans J. D.; Bon V.; Crespi S.; Danowski W.; Browne W. R.; Ehrling S.; Walenszus F.; Wallacher D.; Grimm N.; Többens D. M.; Weiss M. S.; Kaskel S.; Feringa B. L. Cooperative light-induced breathing of soft porous crystals via azobenzene buckling. Nat. Commun. 2022, 13, 1951 10.1038/s41467-022-29149-z. 35414051 PMC9005654

[ref38] Thangavel K.; Walenszus F.; Mendt M.; Bon V.; Kaskel S.; Pöppl A. Monitoring the Local Structure and Magnetic Properties of the Dinuclear Cu_2_-Paddle Wheel Nodes in the Mesoporous Metal-Organic Framework, DUT-49(Cu), upon Adsorption-Induced Breathing Transitions. J. Phys. Chem. C 2023, 127, 8217–8234. 10.1021/acs.jpcc.2c08905.

[ref39] Dippel A.-C.; Liermann H.-P.; Delitz J. T.; Walter P.; Schulte-Schrepping H.; Seeck O. H.; Franz H. Beamline P02.1 at PETRA III for high-resolution and high-energy powder diffraction. J. Synchrotron Radiat. 2015, 22, 675–687. 10.1107/S1600577515002222. 25931084 PMC4416682

[ref40] Ashiotis G.; Deschildre A.; Nawaz Z.; Wright J. P.; Karkoulis D.; Picca F. E.; Kieffer J. The fast azimuthal integration Python library: *pyFAI*. J. Appl. Crystallogr. 2015, 48, 510–519. 10.1107/S1600576715004306. 25844080 PMC4379438

[ref41] Juhás P.; Davis T.; Farrow C. L.; Billinge S. J. L. *PDFgetX3*: a rapid and highly automatable program for processing powder diffraction data into total scattering pair distribution functions. J. Appl. Crystallogr. 2013, 46, 560–566. 10.1107/S0021889813005190.

[ref42] Yang X.; Juhas P.; Farrow C. L.; Billinge S. J. L. xPDFsuite: an end-to-end software solution for high throughput pair distribution function transformation, visualization and analysis, 2015. arXiv:1402.3163. https://arxiv.org/abs/1402.3163.

[ref43] Billinge S. J. L.; Farrow C. L. Towards a robust ad hoc data correction approach that yields reliable atomic pair distribution functions from powder diffraction data. J. Phys.: Condens. Matter 2013, 25, 454202 10.1088/0953-8984/25/45/454202. 24140913

[ref44] Macrae C. F.; Sovago I.; Cottrell S. J.; Galek P. T. A.; McCabe P.; Pidcock E.; Platings M.; Shields G. P.; Stevens J. S.; Towler M.; Wood P. A. *Mercury 4.0*: from visualization to analysis, design and prediction. J. Appl. Crystallogr. 2020, 53, 226–235. 10.1107/S1600576719014092. 32047413 PMC6998782

[ref45] Krause S.; Evans J. D.; Bon V.; Senkovska I.; Coudert F.-X.; Többens D. M.; Wallacher D.; Grimm N.; Kaskel S. The role of temperature and adsorbate on negative gas adsorption transitions of the mesoporous metal-organic framework DUT-49. Faraday Discuss. 2021, 225, 168–183. 10.1039/D0FD00013B. 33118556

[ref46] Lv D.; Chen Y.; Li Y.; Shi R.; Wu H.; Sun X.; Xiao J.; Xi H.; Xia Q.; Li Z. Efficient Mechanochemical Synthesis of MOF-5 for Linear Alkanes Adsorption. J. Chem. Eng. Data 2017, 62, 2030–2036. 10.1021/acs.jced.7b00049.

[ref47] Bao Z.; Alnemrat S.; Yu L.; Vasiliev I.; Ren Q.; Lu X.; Deng S. Adsorption of Ethane, Ethylene, Propane, and Propylene on a Magnesium-Based Metal-Organic Framework. Langmuir 2011, 27, 13554–13562. 10.1021/la2030473. 21942644

