# Supplementary material for: Probing the Limits of Mechanical Stability of the Mesoporous Metal–Organic Framework DUT-76(Cu) by Hydrocarbon Physisorption
Source: ACS Appl Mater Interfaces. 2025 Apr 8;17(16):24096–105. doi: 10.1021/acsami.5c00164 (PMC12022944; doi:10.1021/acsami.5c00164)
Supplement: Supplementary file 1 — am5c00164_si_001.pdf [file am5c00164_si_001.pdf]

Supporting Information for

Probing the limits of mechanical stability of the mesoporous Metal-Organic Framework DUT-76(Cu) by hydrocarbons physisorption

Kai Konowski,[a] Volodymyr Bon,[a] Martin A. Karlsen,[b] Martin Etter,[b] Nadine Bönisch,[a] Ankita De,[a] Stefan Kaskel*[a]*

[a] Chair of Inorganic Chemistry I, Technische Universität Dresden, Bergstraße 66, 01069 Dresden

[b] P02.1 Beamline, PETRA III Synchrotron, DESY, Notkestr. 85, 22607 Hamburg

Chemicals and Methods

Chemicals

All chemicals were purchased from the supplier and used in the purity described in Table S1. Ethanol and tetrahydrofuran were additionally purified in a MBraun MB-SPS 800.

Table S1. List of chemicals used for the synthesis of ligands and MOFs.

Name	CAS	Purity	Supplier
**Acetic acid**	64-19-2	100% p.a.	Roth
**Carbon dioxide**	124-38-9	4.6 50H	Nippon Gases
**Copper(I) iodide**	7681-65-4	98%	Sigma Aldrich
**Copper(II)nitrate trihydrate**	10031-43-3	Emsure	Sigma Aldrich
**4,4'-Diiodobiphenyl**	3001-15-8	99%	Thermo Scientific
**N,N-Dimethylformamide**	68-12-2	99.8%	Thermo Scientific
**Ethanol**	64-17-5	99.8%, SPS purified	Fisher Scientific
**Hydrochloric acid**	7647-01-1	37% p.a.	Honeywell
**Iso-Propyl magnesium chloride**	1068-55-9	2M in THF	Sigma Aldrich
**Magnesium sulfate**	7487-88-9	99%	Grüssing GmbH
**n-Butanol**	71-36-3	99%	Thermo Scientific
**L-Proline**	147-85-3	≥ 99%	Sigma Aldrich
**Potassium carbonate**	584-08-7	≥ 99.5%	Fisher Chemicals
**Potassium hydroxide**	1310-58-3	Reagent grade	Fisher Chemicals
**Sodium hydroxide**	1310-73-2	technical	VWR chemicals
**Tetrahydrofuran**	109-99-9	99.8%, SPS purified	Fisher scientific

Instruments and Methods

Nuclear magnetic resonance (NMR) spectroscopy

Bruker Ascend 300 (300 MHz and 76 MHz for ^{1}H and ^{13}C, respectively). All ^{1}H and ^{13}C NMR spectra are reported in parts per million (ppm) downfield of TMS and were measured relative to the residual signals of the solvents at 5.32 ppm (CH_2Cl_2) and 2.50 ppm (DMSO). Data for ^{1}H NMR spectra are described as following: chemical shift (δ (ppm)), multiplicity (s, singlet; d, doublet; m, multiplet; br, broad signal), coupling constant J (Hz), integration corresponding to amount of H, C or CH. Data for ^{13}C NMR spectra are described spectra relative to the residual signals of the solvents at 54.00 ppm (CH_2Cl_2) and 39.52 ppm (DMSO-d_6) in terms of chemical shift (δ (ppm)) and functionality were derived from DEPT135.

High-resolution mass spectrometry (HRMS)

HRMS was measured with a Waters Xevo G2-XS QTof. The sample of the linker was prepared by dissolving 1 mg of the linker in 1 mL methanol.

Synthesis of Organic Ligands

Synthetic route

Dibutyl *9H*-carbazole-3,6-dicarboxylate (CDC(n-Bu)$_2$) was prepared according to a literature procedure.[47] The linker was synthesized according to a procedure developed by Stoeck et al. (see **Scheme S1**).[34]

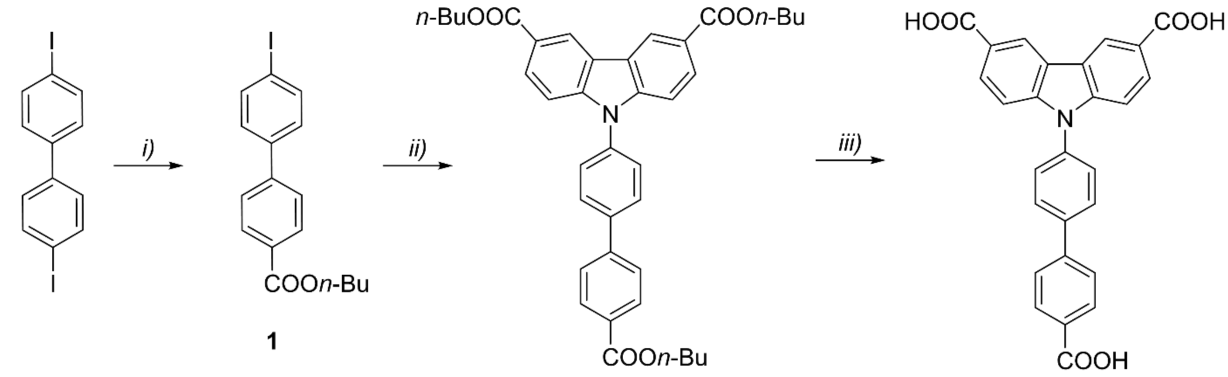

1 **2** **3**

Scheme S1. i) iPrMgCl, CO_2, THF; 2. EtOH, H_2SO_4, 77 %; ii) CuI, K_2CO_3, L-proline, DMSO, 90 °C, 56 %; iii) KOH, THF, MeOH, H_2O, reflux, 85 %.

Synthesis of Butyl-4'-iodo-(1,1'-biphenyl)-4-carboxylate (1)

An oven-dried 500 mL three-neck flask equipped with a glass valve and a dripping funnel, was charged with 4,4‘-diiodobiphenyl (20 g, 49.26 mmol) under argon atmosphere and dissolved in 500 mL dry THF. The solution was then cooled to 0°C using an ice bath. Isopropyl magnesium chloride solution (2M, 25 mL, 50 mmol) was added through the dripping funnel over one hour. The ice bath was removed and the mixture was left to stir for one more hour. Subsequently dry carbon dioxide was bubbled through the solution for one hour and the mixture was stirred under a carbon dioxide atmosphere overnight. The mixture was poured into aqueous hydrochloric acid (2 M, 500 mL) to precipitate the raw acid, which was then filtered off and washed with four times 100 mL water and dried on the filter. Subsequently the raw acid was resuspended in 500 mL of *n*-butanol and 5 mL of concentrated sulfuric acid are added. The mixture was refluxed for 48 h. After cooling down to room temperature the alcohol was removed in vacuo and the solid residue was taken up in 750 mL chloroform. This solution was then washed with 500 mL water. Saturated sodium hydrogencarbonate solution was added to the aqueous phase until the acid was neutralized. The organic phase was dried with magnesium sulfate and the solvent was removed in vacuo. The resulting raw product was purified by flash chromatography (DCM/iso-hexane 1:1), recrystallized from ethylacetate and then used without further purification.

Yield: 14.40 g (38 mmol, 77 %).

^{1}H NMR (300 MHz, DMSO-d$_6$) δ 7.99 (d, J = 8.5 Hz, 2H), 7.81 (d, J = 8.5 Hz, 2H), 7.74 (d, J = 8.5 Hz, 2H), 7.48 (d, J = 8.5 Hz, 2H), 4.25 (t, J = 6.5 Hz, 2H), 1.77 – 1.57 (m, 2H), 1.40 (d, J = 7.5 Hz, 2H), 0.91 (t, J = 7.5 Hz, 3H).

^{13}C NMR and DEPT135 (76 MHz, DMSO-d$_6$) δ 165.45 (C_q), 143.47 (C_q), 138.29 (C_q), 137.83 (CH_{ar}), 129.78 (CH_{ar}), 129.05 (CH_{ar}), 129.00 (C_q), 126.75 (CH_{ar}), 95.08 (C_q), 64.41 (CH_2), 30.23 (CH_2), 18.74 (CH_2), 13.59 (CH_3).

Synthesis of dibutyl 9-(4'-(butoxycarbonyl)-[1,1'-biphenyl]-4-yl)-9H-carbazole-3,6-dicarboxylate (2)

A 250 mL schlenk tube was charged with dibutyl 9H-carbazole-3,6-dicarboxylate (CDC(n-Bu)$_2$) (5.0 g, 13.61 mmol), butyl 4'-iodo-(1,1'-biphenyl)-4-carboxylate (6.21 g, 16.33 mmol), copper(I) iodide (259.2 mg, 1.36 mmol), L-proline (321.6 mg, 2.79 mmol) and potassium carbonate (386 mg, 2.97 mmol). After adding 50 mL DMSO the resulting suspension was degassed in vacuum (0.1 Pa) for 30 min. The flask was then backfilled with argon and heated to 90 °C for 3 d. The reaction mixture was poured into 500 mL water and extracted three times with 750 mL dichloromethane each. The combined organic phases were dried over magnesium sulfate and the solvent was removed in vacuo. The solid residue was purified using flash chromatography (toluene/ethylacetate 7:1). Recrystallization with ethylacetate yielded the pure product.

Yield: 4.71 g (7.6 mmol, 56 %)

^{1}H NMR (300 MHz, CD_2Cl_2) δ 8.95 (d, *J* = 1.2 Hz, 2H), 8.23 - 8.12 (m, 4H), 7.93 (d, *J* = 8.5 Hz, 2H), 7.80 (d, *J* = 8.5 Hz, 2H), 7.69 (d, *J* = 8.5 Hz, 2H), 7.48 (d, *J* = 8.5 Hz, 2H), 4.44 - 4.30 (m, 6H), 1.90 - 1.71 (m, 6H), 1.66 – 1.46 (m, 6H), 1.08 - 0.95 (m, 9H).

^{13}C NMR and DEPT135 (76 MHz, CD_2Cl_2) δ 167.34 (C_q), 166.76 (C_q), 144.69 (2 C_q), 140.69 (C_q), 136.92 (C_q), 130.70 (CH_{ar}), 130.60 (C_q), 129.56 (CH_{ar}), 128.65 (CH_{ar}), 128.02 (CH_{ar}), 127.65 (CH_{ar}), 123.96 (C_q), 123.78 (C_q), 123.41 (CH_{ar}), 110.38 (CH_{ar}), 65.50 (1 CH_2), 65.35(2 CH_2), 31.51 (2 CH_2), 31.38 (1 CH_2), 19.95 (2 CH_2), 19.89 (1 CH_2), 14.18 (2 CH_3), 14.13(1 CH_3).

Synthesis of 9-(4'-carboxy-[1,1'-biphenyl]-4-yl)-9H-carbazole-3,6-dicarboxylic acid (3)

In a 500 mL round bottom flask potassium hydroxide (11.68 g, 208.1 mmol) was dissolved in 120 mL water. Dibutyl 9-(4'-(butoxycarbonyl)-[1,1'-biphenyl]-4-yl)-9H-carbazole-3,6-dicarboxylate (2) (4.30 g, 6.94 mmol), 150 mL THF and 150 mL methanol were added. The mixture was stirred under reflux overnight and is cooled to room temperature. The solvents are removed in vacuo and hydrochloric acid (1 M, 200 mL) is added to precipitate the linker as a carboxylic acid. The precipitate is filtered off and washed with water until the filtrate was not acidic anymore. The product was then dried in vacuo.

Yield: 2.65 g (5.9 mmol, 85 %)

^{1}H NMR (300 MHz, DMSO-d_6) δ 12.89 (br. s, 3H), 8.99 (d, *J* = 1.2 Hz, 2H), 8.16 – 8.02 (m, 6H), 7.95 (d, *J* = 8.6 Hz, 2H), 7.82 (d, *J* = 8.6 Hz, 2H), 7.52 (d, *J* = 8.6 Hz, 2H).

^{13}C NMR and DEPT135 (76 MHz, DMSO-d_6) δ 167.61 (C_q), 167.08 (C_q), 143.41 (C_q), 143.21 (C_q), 139.06 (C_q), 135.81 (C_q), 130.11 (CH_{ar}), 130.05 (Cq), 128.91 (CH_{ar}), 128.34 (CH_{ar}), 127.54 (CH_{ar}), 127.06 (CH_{ar}), 123.49 (C_q), 123.06 (CH_{ar}), 122.61 (C_q), 110.01 (CH_{ar}).

ESI-TOF-HRMS (m/z): Calculated for [M -H^+]: 450.0978; found: [M -H^+]-: 450.0981.

Synthesis and Characterization of DUT-76(Cu)

Synthesis of microcrystalline DUT-76(Cu) powder

DUT-76(Cu) was synthesized as a microcrystalline powder by solvothermal synthesis according to a published procedure.[34] In a 100 mL screw cap bottle 361.1 mg 9-(4'-carboxy-[1,1'-biphenyl]-4-yl)-9H-carbazole-3,6-dicarboxylic acid (3) (0.80 mmol, 1 eq.), 309.2 mg Copper(II) nitrate trihydrate (1.28 mmol, 1.6 eq.) were dissolved in 72 mL of mixture dry DMF and dry EtOH (1:1) and 3.2 mL acetic acid (3.36 g, 56.00 mmol, 70 eq.) was added. The compounds were dissolved by ultrasonication for 5 minutes and the solution was then transferred into 8 Pyrex® tubes and heated to 80 °C for 2 d. The blue crystals were washed with fresh solvent mixture (dry DMF and dry EtOH, 1:1) 5 times and afterwards exchanged with dry acetone. Desolvation of DUT-76(Cu) was then achieved using a supercritical point dyer and resulted in yield MOF powder.

Yield: 262 mg (0.5 mmol, 61%)

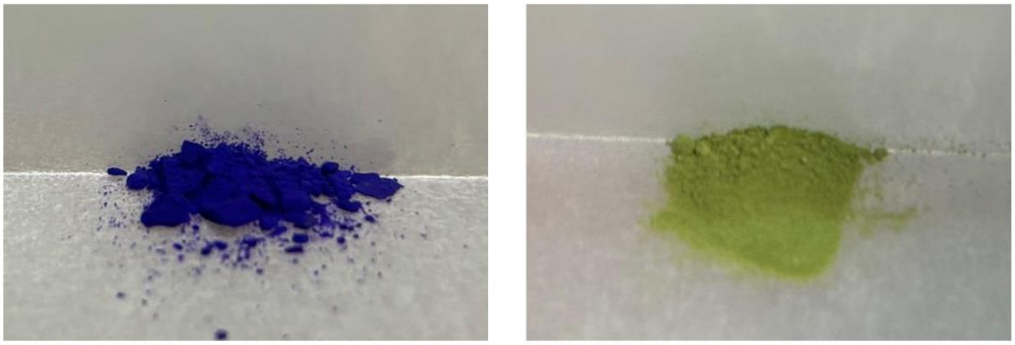

Figure S1. Microcrystalline powder of DUT-76(Cu) activated (left) and decomposed after multi cycle Powder X-ray diffraction (PXRD) measurement (right).

In situ data of DUT-76(Cu)

Propane

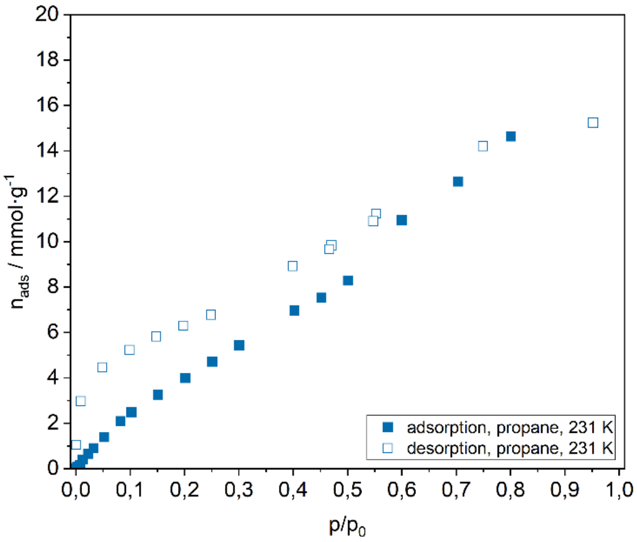

Figure S2. Isotherm of DUT-76(Cu) with propane measured at 231 K in parallel to *in situ* PXRD measurements.

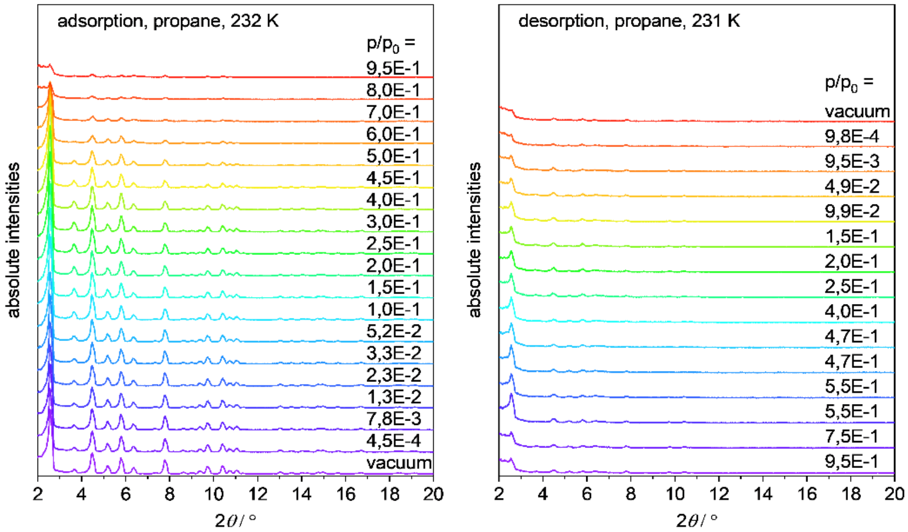

Figure S3. *In situ* PXRD patterns (λ = 0.15405 nm), parallel to adsorption (left) and desorption (right) of propane at 231 K.

Propylene

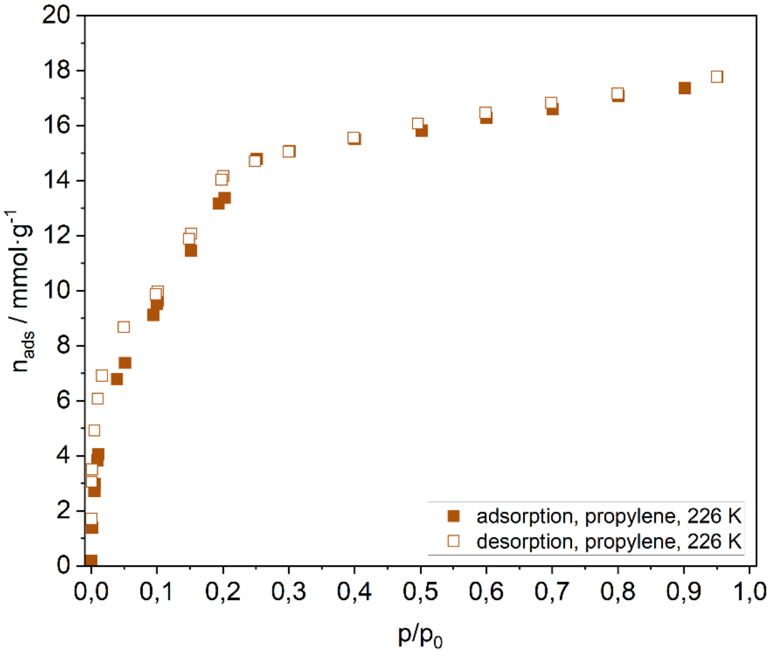

Figure S4. Isotherm of DUT-76(Cu) with propylene measured at 226 K alongside *in situ* PXRD measurement.

n-Butane

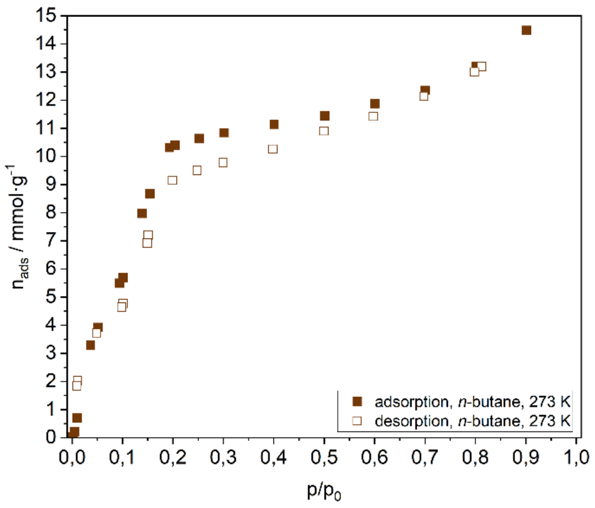

Figure S5. Isotherm of DUT-76(Cu) with *n*-butane measured at 273 K alongside *in situ* PXRD measurement.

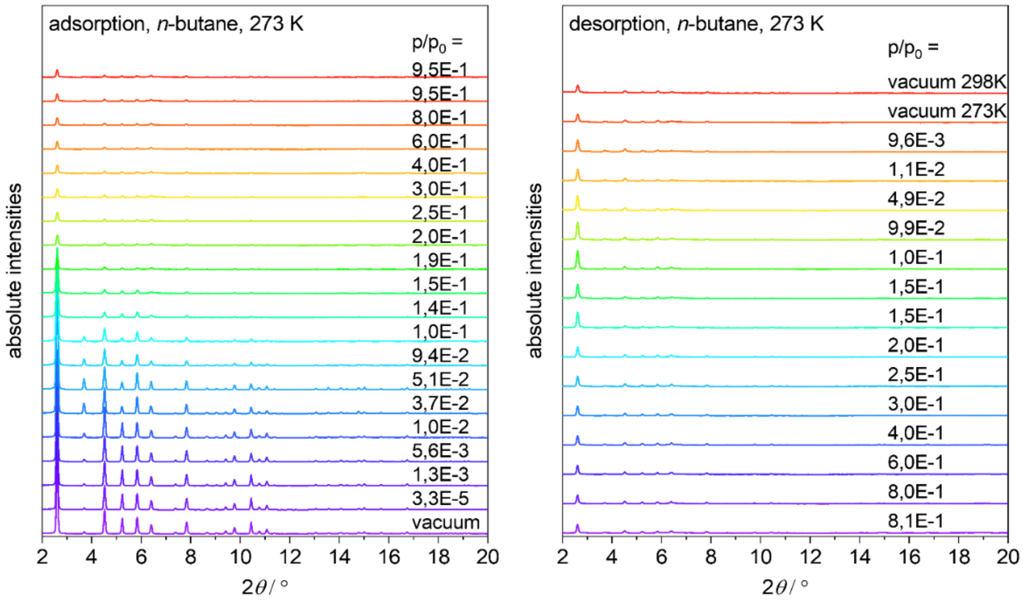

Figure S6. *In situ* PXRD patterns (λ = 0.15405 nm), in parallel to adsorption (left) and desorption (right) of *n*-butane at 273 K.
